# Supplementary material for: Efficient sequential Bayesian inference for state-space epidemic models using ensemble data assimilation
Source: PLoS Comput Biol. 2026 May 18;22(5):e1014301. doi: 10.1371/journal.pcbi.1014301 (PMC13221153; doi:10.1371/journal.pcbi.1014301)
Supplement: S1 Text — (PDF) [file pcbi.1014301.s001.pdf]

Supplementary Materials: Efficient sequential Bayesian inference for state-space epidemic models using ensemble data assimilation

Dhorasso Temfack and Jason Wyse
School of Computer Science and Statistics, Trinity College Dublin, Ireland

A Transition density and observation error variance

A.1 Transition density of the SEIR model

We discretize the continuous-time SEIR dynamics using a forward Euler scheme for the compartmental states $(S_t, E_t, I_t, R_t, Z_t)$ and an Euler–Maruyama scheme for the stochastic transmission rate $\log(\beta_t)$. The compartments are propagated deterministically according to

$$\begin{cases} S_t = S_{t-1} - \Delta t\, \beta_{t-1} \dfrac{S_{t-1} I_{t-1}}{N}, \\ E_t = E_{t-1} + \Delta t \left(\beta_{t-1} \dfrac{S_{t-1} I_{t-1}}{N} - \alpha E_{t-1} \right), \\ I_t = I_{t-1} + \Delta t \,(\alpha E_{t-1} - \gamma I_{t-1}), \\ R_t = R_{t-1} + \Delta t\, \gamma I_{t-1}, \\ Z_t = \alpha E_{t-1} \Delta t. \end{cases} \tag{1}$$

where Δt represents the discretization step (one day in our implementation). Formally, this deterministic update can be represented as a Dirac delta distribution in the transition density:

$$p(S_t, E_t, I_t, R_t, Z_t | x_{t-1}, \theta) = \delta_{f(x_{t-1}, \theta)}(S_t, E_t, I_t, R_t, Z_t), \tag{2}$$

where $f(\cdot)$ is the Euler update map in (1). The stochastic log-transmission rate is propagated as

$$\log(\beta_t) = \log(\beta_{t-1}) + \nu_\beta \sqrt{\Delta t}\, \epsilon_t, \quad \epsilon_t \sim \mathcal{N}(0, 1), \tag{3}$$

so that the full transition density for the latent state x_t is given by

$$p(x_t | x_{t-1}, \theta) = \delta_{f(x_{t-1}, \theta)}(S_t, E_t, I_t, R_t, Z_t)\, \mathcal{N}(\log(\beta_t);\ \log(\beta_{t-1}),\ \nu_\beta^2). \tag{4}$$

A.2 Kalman gain and observation error variance

We consider the stochastic process $(x_t, y_t)_{t \geq 0}$ defined in Section 2 of the main text, such that $y_t | x_t \sim p(y_t | x_t)$ with finite conditional mean $\mathrm{E}[y_t | x_t] = H x_t$. Then

$$\begin{aligned} \mathrm{E}\Big[\big(x_t - \mathrm{E}[x_t]\big) \big(y_t - \mathrm{E}[y_t | x_t]\big) \Big] &= \mathrm{E}\Big[\mathrm{E}\big[\big(x_t - \mathrm{E}[x_t]\big) \big(y_t - \mathrm{E}[y_t | x_t]\big) \,\big|\, x_t \big] \Big] \\ &= \mathrm{E}\Big[\big(x_t - \mathrm{E}[x_t]\big) \underbrace{\mathrm{E}\big[y_t - \mathrm{E}[y_t | x_t] \,\big|\, x_t \big]}_{=0} \Big] = 0. \end{aligned} \tag{5}$$

Equation (5) implies that the deviations of the latent state are uncorrelated with the observation residuals, where the residuals are defined relative to their conditional mean. This result

generalizes Lemma 1 of Ebeigbe et al. (2020) to any observation distribution with a finite conditional mean. We stress that this property does not imply that a linear Kalman gain is optimal in a nonlinear setting. Nevertheless, it justifies using a linear gain K_t to update the state estimate:

$$\begin{aligned} K_t &= \mathrm{Cov}\big[x_t, y_t\big]\big(\mathrm{Var}[y_t]\big)^{-1} \\ &= \mathrm{Cov}\big[x_t, \mathrm{E}[y_t|x_t] + y_t - \mathrm{E}[y_t|x_t]\big]\big(\mathrm{Var}[y_t]\big)^{-1} \\ &= \mathrm{Cov}\big[x_t, \mathrm{E}[y_t|x_t]\big]\big(\mathrm{Var}[y_t]\big)^{-1} \\ &= \mathrm{Cov}\big[x_t, \mathrm{E}[y_t|x_t]\big]\Big(\mathrm{Var}\big[\mathrm{E}[y_t|x_t]\big] + \mathrm{E}\big[\mathrm{Var}[y_t|x_t]\big]\Big)^{-1} \\ &\approx \widehat{\Sigma}_{t|t-1} H^\top \left(H \widehat{\Sigma}_{t|t-1} H^\top + \underbrace{\frac{1}{N_x} \sum_{i=1}^{N_x} \mathrm{Var}\big[y_t | x_t^{(f,i)}\big]}_{V_t^{N_x}} \right)^{-1} . \end{aligned} \tag{6}$$

The third line follows because the residual $(y_t - \mathrm{E}[y_t|x_t])$ is uncorrelated with x_t, as shown in (5). The fourth line is a direct application of the law of total variance and the last line is an ensemble approximation of the Kalman gain, where $\widehat{\Sigma}_{t|t-1}$ is the forecast ensemble covariance.

B Bootstrap Particle Filter

An unbiased estimate of the incremental likelihood can be obtained using a particle filter. Algorithm B1 outlines the Bootstrap Particle Filter (BPF), which is the version employed in this paper within the standard SMC^2 framework. Algorithm B2 describes the stratified resampling procedure, which is used in both SMC^2 and eSMC^2 implementations.

Algorithm B1 Bootstrap Particle Filter (BPF)

Operations involving index i must be performed for $i = 1, \dots, N_x$.
The indices $a_t^{1:N_x}$ define the ancestral state particles at time t after the resampling.
Inputs: Observation: $y_{1:T}$, Number of particles: N_x, Inital state distribution: $p(x_0)$, Parameter vector θ.
Output: Particles set: $\{x_{0:t}^i, w_{0:t}^i\}_{i=1}^{N_x}$, marginal likelihood $\widehat{p}_{\mathrm{bpf}}^{N_x}(y_{1:t}|\theta)$

```
Sample initial particles : x_0^i ~ p(x_0)
Compute weights: w_0^i = 1,   W_0^i = 1/N_x
for t = 1 to T do
    Sample new indices: a_t^{1:N_x} ~ Resample(W_{t-1}^{1:N_x})        ▷ Algorithm B2
    Propagate states x_t^i ~ p(·|x_{0:t-1}^{a_t^i}, θ)
    Compute weights and normalize: w_t^i = p(y_t|x_t^i, θ) ,   W_t^i = w_t^i / Σ_{j=1}^{N_x} w_t^j
    Compute the incremental likelihood: p̂_bpf^{N_x}(y_t|y_{1:t-1}, θ) = (1/N_x) Σ_{i=1}^{N_x} w_t^i
end for
Compute marginal likelihood: p̂_bpf^{N_x}(y_{1:T}|, θ) = Π_{t=1}^{T} p̂_bpf^{N_x}(y_t|y_{1:t-1}, θ).
```

Algorithm B2 Stratified Resampling

Inputs: Normalized weights $W^{1:N_x}$, number of particles N_x.
Output: Resampled indices $a^{1:N_x}$.

```
Compute cumulative weights: C_i = sum_{j=1}^{i} W^j for i = 1, ..., N_x
for k = 1, ..., N_x do
    Sample u_k ~ Uniform((k-1)/N_x, k/N_x)
end for
Set i ← 1
for k = 1, ..., N_x do
    while u_k > C_i do
        i ← i + 1
    end while
    a^k ← i
end for
```

C Additional results from simulated experiments

This section presents supplementary figures supporting the results discussed in the main text. Figures C.1–C.3 show the filtered estimated trajectories of the unobserved SEIR compartments, while Figures C.4–C.6 display the posterior pairwise parameter distributions obtained from SMC^2 and eSMC^2 for Examples 1, 2 and 3.

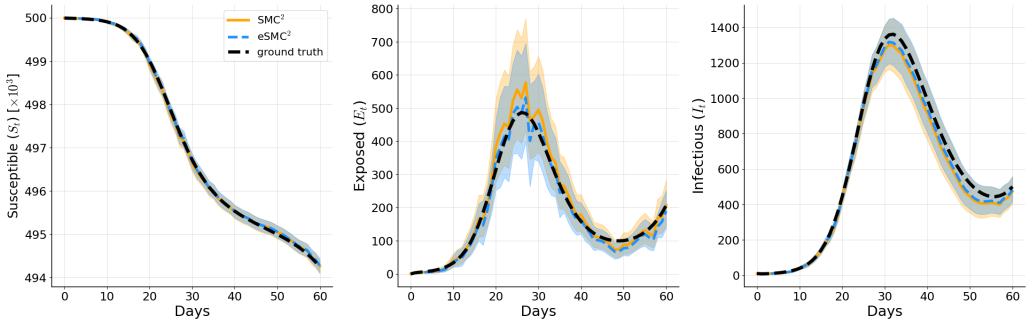

Figure C.1: **Example 1: Unobserved states of the SEIR model.** Filtering mean and 95% credible interval are shown for each compartments. The black dashed line indicates the ground truth.

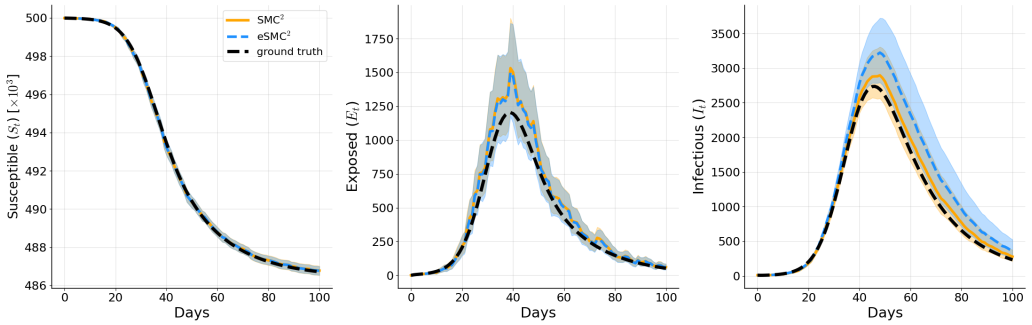

Figure C.2: **Example 2: Unobserved states of the SEIR model.** Filtering mean and 95% credible interval are shown for each compartments. The black dashed line indicates the ground truth.

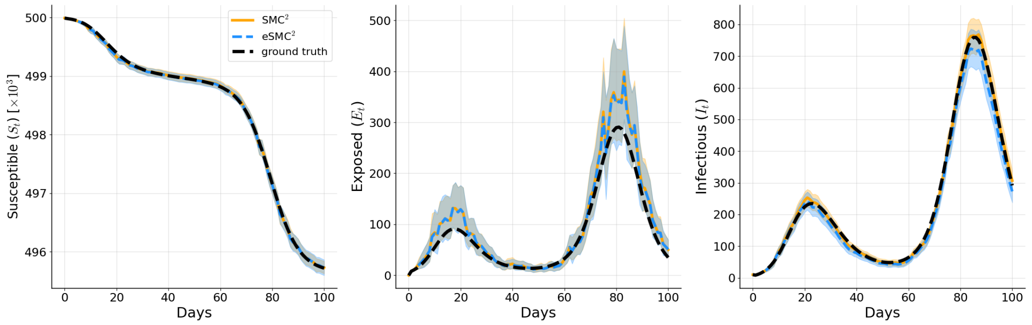

Figure C.3: **Example 3: Unobserved states of the SEIR model.** Filtering mean and 95% credible interval are shown for each compartments. The black dashed line indicates the ground truth.

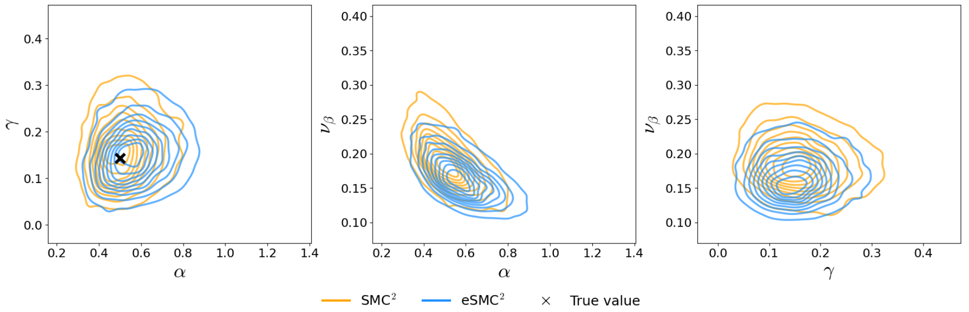

Figure C.4: **Posterior distributions of model parameters for Example 1.** Each subplot shows the pairwise marginal densities of (α, γ), (α, ν_β), and (γ, ν_β). Contours represent the combined posterior from five runs of SMC^2 (orange) and eSMC^2 (blue), with black crosses indicating the true parameter values.

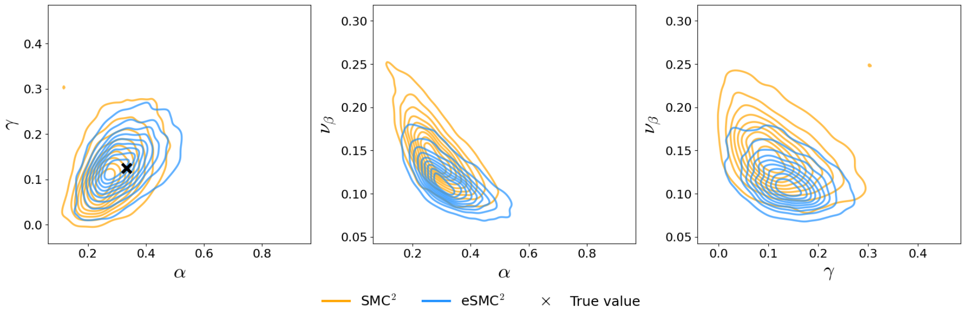

Figure C.5: **Posterior distributions of model parameters for Example 2.** Each subplot shows the pairwise marginal densities of (α, γ), (α, ν_β), and (γ, ν_β). Contours represent the combined posterior from five runs of SMC^2 (orange) and eSMC^2 (blue), with black crosses indicating the true parameter values.

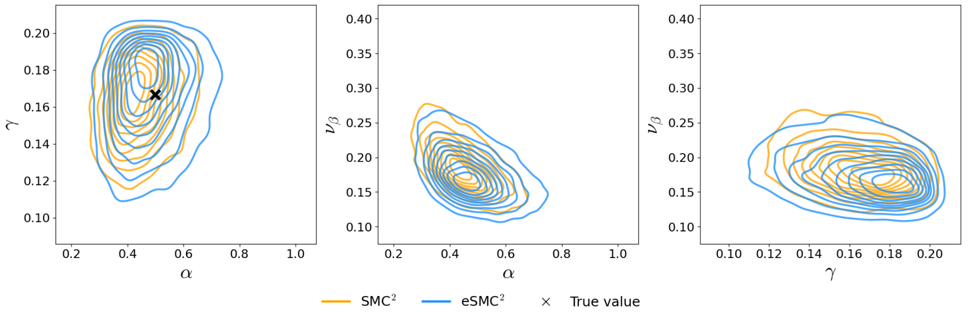

Figure C.6: **Posterior distributions of model parameters for Example 3.** Each subplot shows the pairwise marginal densities of (α, γ), (α, ν_β), and (γ, ν_β). Contours represent the combined posterior from five runs of SMC^2 (orange) and eSMC^2 (blue), with black crosses indicating the true parameter values.

D Sensitivity to ensemble size and prior

Table D.1 reports the results of eSMC^2 for different values of the number of state particles (N_x), with the number of parameter particles fixed at $N_\theta = 1000$. Across both examples, increasing N_x leads to higher computational cost but only marginal improvements in parameter estimate, suggesting that moderate ensemble sizes (around $N_x = 150 - 300$) offer a good trade-off between accuracy and efficiency.

Table D.1: **eSMC^2 sensitivity analysis across N_x.** Posterior mean and standard deviation (in parentheses) are shown for each estimated parameter. CPU time is reported in seconds.

N_x	CPU	α	γ	ν_β
Example 1				
50	271	0.589 (0.089)	0.161 (0.056)	0.174 (0.026)
100	302	0.580 (0.126)	0.161 (0.048)	0.169 (0.030)
200	430	0.560 (0.126)	0.142 (0.055)	0.178 (0.038)
400	986	0.554 (0.107)	0.162 (0.055)	0.171 (0.027)
800	1320	0.558 (0.111)	0.154 (0.055)	0.178 (0.030)
Truth		0.5	0.142	-
Example 2				
50	360	0.294 (0.055)	0.123 (0.045)	0.129 (0.019)
100	401	0.311 (0.085)	0.132 (0.050)	0.123 (0.023)
200	627	0.304 (0.068)	0.131 (0.041)	0.120 (0.020)
400	1035	0.302 (0.076)	0.143 (0.052)	0.129 (0.027)
800	1770	0.314 (0.080)	0.116 (0.056)	0.131 (0.027)
Truth		0.333	0.125	-
Example 3				
50	304	0.479 (0.075)	0.167 (0.017)	0.169 (0.022)
100	379	0.484 (0.086)	0.169 (0.019)	0.166 (0.025)
200	615	0.479 (0.078)	0.173 (0.016)	0.162 (0.021)
400	1000	0.483 (0.102)	0.166 (0.021)	0.176 (0.033)
800	1921	0.485 (0.094)	0.166 (0.021)	0.179 (0.032)
Truth		0.5	0.167	-

In Figure D.1, we examine how changes in the reporting fraction ρ affect the recovery of key model parameters. The simulated data were generated under the same settings as in Example 1, but with reporting probabilities $y_t \sim \text{Poiss}(\rho Z_t)$ for $\rho \in \{0.5, 0.6, 0.7, 0.8, 0.9, 1\}$. During inference, we assumed that ρ was known and matched the value used in data generation. The results show that, although the magnitudes of some estimates, especially β_t and the early values of γ and ν_β, vary with ρ, the overall temporal patterns and qualitative behaviour remain largely robust across reporting scenarios. This indicates that the framework can reliably recover the main dynamic features of the system when the reporting fraction is correctly specified.

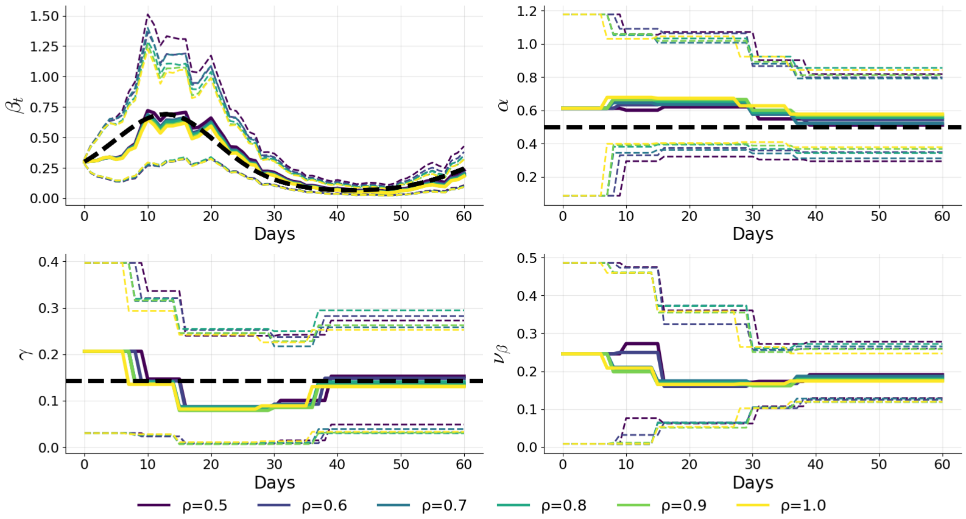

Figure D.1: **Filtered estimate estimates of β_t, α, γ, and ν_β.** Results are obtained using the eSMC^2 algorithm on Example 1 under different fixed reporting fractions ρ. Solid lines denote posterior medians and dashed lines the associated 95% credible intervals; black dashed lines indicate the ground truth value.

We also assessed the impact of using non-informative priors by assigning $\alpha, \nu_\beta \sim \mathcal{U}(0,1)$ and $\gamma \sim \mathcal{U}(0,1)$ for Examples 1 and 2, $\gamma \sim \mathcal{U}(0.1, 0.2)$ for Example 3, and fitting the model to simulated data. With flat priors, the posterior distribution is largely determined by the likelihood, causing the posterior mode to align closely with the maximum likelihood estimate. Figures D.2, D.3 and D.4 summarize the results for Examples 1, 2 and 3, respectively, showing the evolution of the filtered parameter trajectories and the final marginal and joint posterior distributions.

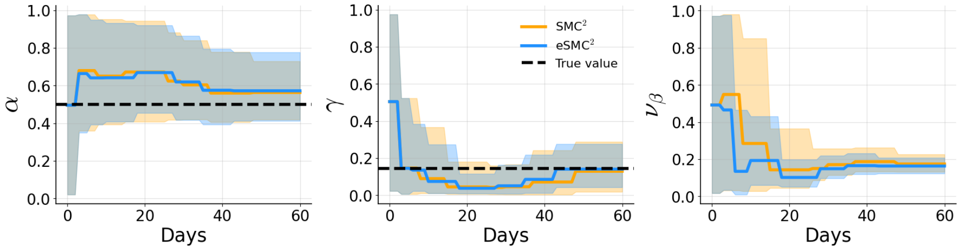

Figure D.2: **Example 1: Sensitivity of posterior estimates to prior specification.** filtered means with 95% credible intervals (top) and pairwise marginal distributions at the final time step ($T = 60$) (bottom). The black dashed lines denote the true parameter values.

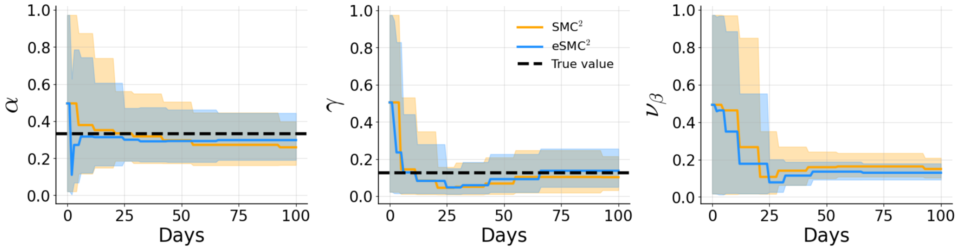

Figure D.3: **Example 2: Sensitivity of posterior estimates to prior specification.** filtered means with 95% credible intervals (top) and pairwise marginal distributions at the final time step ($T = 100$) (bottom). The black dashed lines denote the true parameter values.

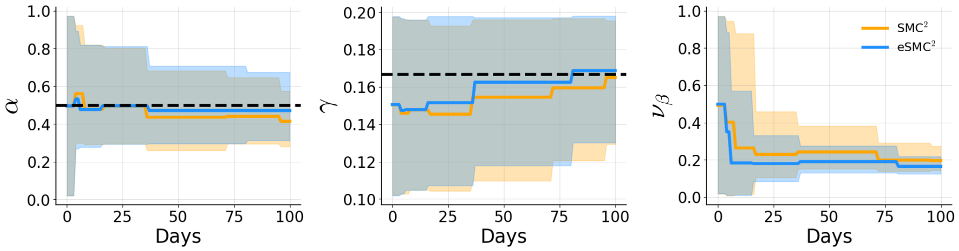

Figure D.4: **Example 3: Sensitivity of posterior estimates to prior specification.** filtered means with 95% credible intervals (top) and pairwise marginal distributions at the final time step ($T = 100$) (bottom). The black dashed lines denote the true parameter values.

In both examples, the filtered means for α and γ quickly converge toward the true parameter values, and the 95% credible intervals narrow as more data are assimilated, indicating progressive information gain from the likelihood. At the final time step, the marginal posterior densities and contour plots reveal compact, unimodal surfaces centered near the true values. The parameters α and γ are particularly well recovered. However, the posterior of ν_β exhibits a slight shift relative to SMC^2. Crucially, the corresponding contour plots show no qualitative change in the joint-dependence structure, that is, eSMC^2 does not produce different correlations or spurious multimodality.

E Comparison with the Liu and West filter

To further evaluate the performance of eSMC^2, we compare it with the well-established particle filtering approach of Liu & West (2001), commonly known as the Liu and West filter. This algorithm provides a benchmark for joint sequential state and parameter estimation within state-space models. It extends the standard particle filter by augmenting the state vector with static parameters and applying kernel density shrinkage to mitigate parameter degeneracy. The algorithm generates samples $\{x_t^{(i)}, \theta_t^{(i)}\}_{i=1}^{N_x} \sim p(x_t, \theta|y_{1:t})$.

At time $t-1$, let $\{x_{t-1}^{(i)}, \theta_{t-1}^{(i)}\}_{i=1}^{N_x}$ denote a set of particles with associated normalized weights $\{W_{t-1}^{(i)}\}_{i=1}^{N_x}$ that collectively approximate the joint posterior distribution $p(x_{t-1}, \theta|y_{1:t-1})$. Upon receipt of a new observation y_t, the aim is to update this representation to approximate the posterior $p(x_t, \theta|y_{1:t})$. This distribution can be written recursively as

$$\begin{aligned} p(x_t, \theta|y_{1:t}) &\propto p(y_t|x_t, \theta)\, p(x_t, \theta|y_{1:t-1}) \\ &\propto p(y_t|x_t, \theta)\, p(x_t|y_{1:t-1}, \theta)\, p(\theta|y_{1:t-1}). \end{aligned} \tag{7}$$

The Liu and West filter extends the standard particle filter by incorporating parameter learning through kernel density shrinkage, thereby mitigating the degeneracy problem that arises when static parameters are treated as fixed latent states. At each time step, the parameter posterior distribution is approximated by a Gaussian mixture:

$$p(\theta|y_{1:t-1}) \approx \sum_{i=1}^{N_x} W_{t-1}^{(i)} \mathcal{N}\big(\theta;\, \lambda\, \theta_{t-1}^{(i)} + (1-\lambda)\, \bar{\theta}_{t-1},\, h^2 V_{t-1}\big), \tag{8}$$

where $\bar{\theta}_{t-1}$ and V_{t-1} denote the empirical mean and covariance of the posterior samples $\{\theta_{t-1}^{(i)}, W_{t-1}^{(i)}\}_{i=1}^{N_x}$ at time $t-1$.

The shrinkage parameter λ is defined as $\lambda = \sqrt{1-h^2}$, where $h^2 = 1 - \left(\frac{3\delta-1}{2\delta}\right)^2$ is the kernel smoothing parameter. This shrinkage step centers each kernel component around the global mean $\bar{\theta}_{t-1}$ and ensures that the mixture variance matches the posterior variance, thereby preventing over-dispersion of the particle cloud. Following Liu & West (2001), we set $\delta = 0.99$ and used $N_x = 20{,}000$ particles. The prior specifications are identical to those described in Section 3 of the main text.

Figure E.1 compares filtered estimates obtained with the Liu and West filter and eSMC^2, along with the final posterior distributions from a long PMCMC run (10^4 iterations), displayed as boxplots at $T = 60$. Both filtering methods track the parameter trajectories, but differences arise in accuracy and uncertainty quantification. The Liu and West filter consistently overestimates the recovery rate γ, likely due to kernel-based shrinkage introducing bias in the joint estimation of static parameters. It also produces wider and less stable credible bands, reflecting increased Monte Carlo variability and weight degeneracy. In contrast, eSMC^2 yields posterior means closer to the true values with narrower, more coherent uncertainty intervals, highlighting its improved stability and accuracy in sequential updating. The PMCMC boxplots serve as a gold-standard reference: eSMC^2 credible intervals align closely with the PMCMC posterior at $T = 60$, whereas the Liu and West filter shows more pronounced discrepancies, particularly for γ.

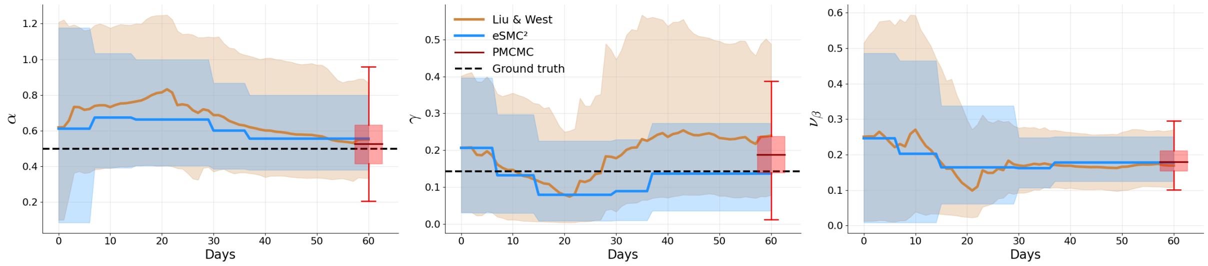

Figure E.1: **Comparison between the Liu and West filter, eSMC^2, and PMCMC for simulated data in Example 1.** Filtered means and 95% credible intervals over time for parameters α, γ, and ν_β. Boxplots at $T = 60$ display the marginal posterior distributions obtained from the PMCMC at time 60.

F Additional results on the mpox dynamics

This section presents supplementary results supporting the analyses in the main text. We first illustrate inference under a Poisson observation model, then present additional results under a Negative Binomial observation model, and finally report forecasting performance metrics used to compare eSMC^2 with a simpler autoregressive baseline.

Inference under Poisson observation variance

Figure F.1 shows estimated weekly incidence and the effective reproduction number for the 2022 U.S. mpox outbreak assuming a Poisson observation model. The filtering procedure captures the overall epidemic trajectory under this simplified variance assumption.

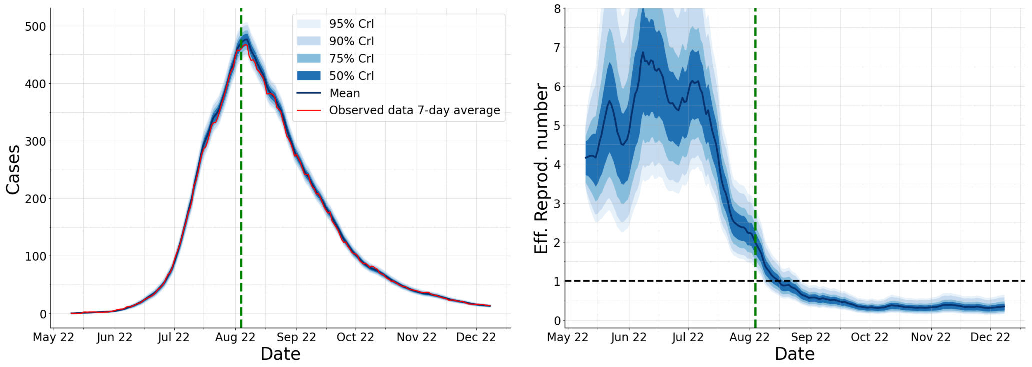

Figure F.1: **Inference of weekly incidence and effective reproduction number under Poisson observation variance.** Filtering mean and 95% credible intervals are shown for reported mpox incidence and estimated R_t.

Additional results under Negative Binomial observation variance

Figure F.2 presents the filtered estimates of the latent state obtained under a Negative Binomial observation model. Figure F.3 presents filtered estimates of the model parameters obtained using eSMC^2 and SMC^2.

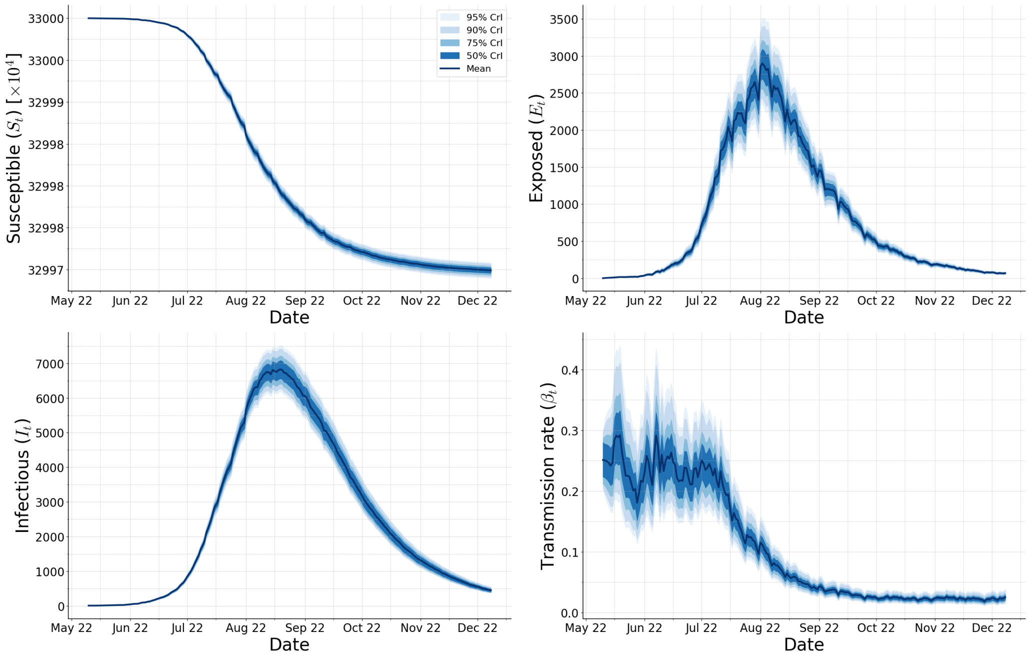

Figure F.2: **Unobserved SEIR states under Negative Binomial observation variance.** Filtering mean and 95% credible intervals are shown for S_t, E_t, I_t, and β_t.

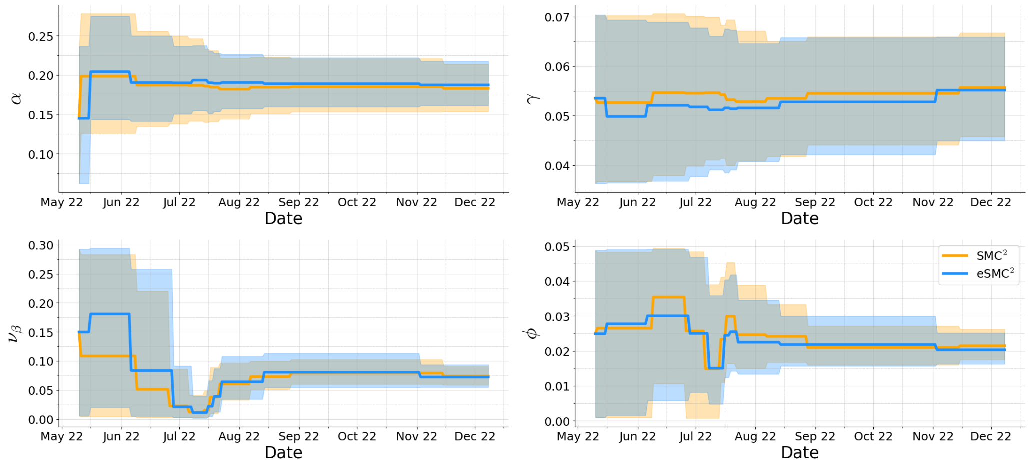

Figure F.3: **Comparison of filtered parameter estimates under Negative Binomial observation variance.** Filtered means and 95% credible intervals obtained with SMC^2 and eSMC^2 are shown.

Autoregressive baseline model for comparison

For reference, we implemented a discrete-time autoregressive model of order p:

$$y_t = a + \sum_{k=1}^{p} b_k y_{t-k} + \varepsilon_t,$$

where Y_t is the observed incidence at time t, a is an intercept, b_k are autoregressive coefficients, and $\varepsilon_t \sim \mathcal{N}(0, \sigma^2)$ represents the innovation term. Predictive distributions were generated by

iteratively simulating forward trajectories conditional on the last p observed values. This baseline captures short-term temporal dependence but does not explicitly model latent transmission dynamics.

Forecast evaluation metrics

Let F_{T+h} denote the predictive distribution at horizon $T+h$ and y_{T+h} the observed incidence. Forecast performance was evaluated using the following metrics:

- **Mean Absolute Error (MAE)**:

$$\text{MAE} = \frac{1}{H}\sum_{h=1}^{H} |y_{T+h} - \hat{m}_{T+h}|,$$

  where H is the forecast horizon and $\hat{m}_{T+h}$ is the predictive median.

- **Weighted Interval Score (WIS)**: To jointly assess probabilistic calibration and sharpness, we compute WIS using central $(1-\alpha)$ prediction intervals (Bracher et al. 2021). Let $l_{\alpha,T+h}$ and $u_{\alpha,T+h}$ be the lower and upper bounds of the $(1-\alpha)$ interval from F_{T+h}. The interval score at horizon $T+h$ is

$$\text{IS}_\alpha(F_{T+h}, y_{T+h}) = (u_{\alpha,T+h} - l_{\alpha,T+h}) + \tfrac{2}{\alpha}(l_{\alpha,T+h} - y_{T+h})\mathbf{1}\{y_{T+h} < l_{\alpha,T+h}\} + \tfrac{2}{\alpha}(y_{T+h} - u_{\alpha,T+h})\mathbf{1}\{y_{T+h} > u_{\alpha,T+h}\}$$

  The WIS combines K intervals and the absolute error of the median:

$$\text{WIS} = \frac{1}{H}\sum_{h=1}^{H}\left[\frac{1}{K+1/2}\left(w_0|y_{T+h} - \hat{m}_{T+h}| + \sum_{k=1}^{K} w_k \text{IS}_{\alpha_k}(F_{T+h}, y_{T+h})\right)\right],$$

  where $w_0 = 1/2$ and $w_k = \alpha_k/2$ are the weights of individual interval scores.

- **Empirical 95% prediction interval coverage**: To measure the proportion of observed outcomes that fall within the model's 95% predictive interval, defined as

$$\text{Coverage}_{95} = \frac{1}{H}\sum_{h=1}^{H}\mathbf{1}\{y_{T+h} \in [l_{0.95,T+h}, u_{0.95,T+h}]\},$$

  where $l_{0.95,T+h}$ and $u_{0.95,T+h}$ are the lower and upper bounds of the 95% prediction interval at horizon $T+h$.

Long-term forecast performance using eSMC^2

Figure F.4 presents forecasts for the final outbreak phase. The model captures the overall declining trend, though short-term daily fluctuations are less well predicted. Predictions are well calibrated, with 95% posterior credible intervals covering all out-of-sample observations. These results confirm that eSMC^2 produces reliable probabilistic forecasts for real-time epidemic monitoring.

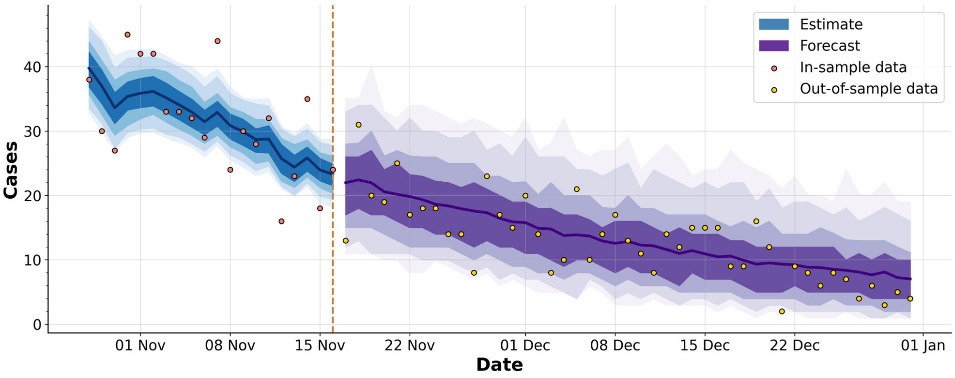

Figure F.4: **Long-term forecast performance with eSMC^2.** Forecast distributions with 50%, 75%, 90%, and 95% credible intervals are shown in purple. In-sample observations (red) occur before the forecast start date (vertical dashed line), while out-of-sample observations (yellow) are used for validation.

References

Bracher, J., Ray, E. L., Gneiting, T. & Reich, N. G. (2021), 'Evaluating epidemic forecasts in an interval format', *PLoS computational biology* **17**(2), e1008618.

Ebeigbe, D., Berry, T., Schiff, S. J. & Sauer, T. (2020), 'Poisson Kalman filter for disease surveillance', *Physical review research* **2**(4), 043028.

Liu, J. & West, M. (2001), Combined parameter and state estimation in simulation-based filtering, *in* 'Sequential Monte Carlo methods in practice', Springer, pp. 197–223.
